# Clonal parental effects on competitive interactions between two duckweeds

**DOI:** 10.3389/fpls.2025.1587907

**Published:** 2025-07-02

**Authors:** Lin-Xuan He, Yu Jin, Xiao-Mei Zhang, Fang-Li Luo, Wei Xue, Jing-Pin Lei, He Liu, Fei-Hai Yu

**Affiliations:** ^1^ Institute of Wetland Ecology and Clone Ecology/Zhejiang Provincial Key Laboratory of Evolutionary Ecology and Conservation, Taizhou University, Taizhou, Zhejiang, China; ^2^ Research Institute of Forestry, Chinese Academy of Forestry, Beijing, China; ^3^ School of Ecology and Nature Conservation, Beijing Forestry University, Beijing, China

**Keywords:** clonal plants, competitiveness, interspecific interactions, maternal effect, nutrient availability, transgenerational plasticity

## Abstract

**Introduction:**

Parental environments can influence offspring fitness via clonal (asexual) propagation, and such clonal parental effects may vary among plant species and depend on offspring environments as well. Consequently, clonal parental effects may alter competitive interactions between plant species, and such impacts may vary with offspring environments.

**Methods:**

We conducted a two-phase experiment with two clonal floating duckweeds, *Spirodela polyrhiza* and *Lemna minor*. In the parental phase, *S. polyrhiza* and *L. minor* were grown separately under two distinct nutrient conditions and produced offspring ramets. In the offspring phase, the ramets produced from the parental phase were grown with or without a heterospecific neighbor under the same two nutrient conditions.

**Results and discussion:**

In the first phase, parent ramets of both species produced more biomass and offspring ramets under high nutrient availability than under low. In the second phase, nutrient availability experienced by the parents significantly affected the competitive ability of offspring in both species. Specifically, the offspring of *L. minor* suppressed those of *S. polyrhiza* more strongly when the parent of *L. minor* had been grown under high than low nutrient availability, although such clonal parental effects did not vary with nutrient availability experienced by the offspring. In contrast, the offspring of *S. polyrhiza* suppressed those of *L. minor* more strongly when the parent of *S. polyrhiza* had been grown under high rather than at low nutrient availability, but this effect occurred only under high nutrient availability for the offspring and diminished under low nutrient availability. These results suggest that clonal parental effects can influence competitiveness of plants and may vary depending on offspring environments. Our findings highlight the potential role of clonal parental effects in regulating interspecific interactions, which may further influence species composition and productivity of plant communities.

## Introduction

In natural environments, phenotypic variation of a plant species is influenced by both its genetic information and non-genetic (environmental) factors (Ellers et al., 2011; Westneat et al., 2019; Wang et al., 2020). Additionally, not only does the environment experienced by a plant individual influence its phenotype, but the environment experienced by its parent(s) also contributes to shaping it (Galloway, 2005; Badyaev and Uller, 2009; Latzel et al., 2023). Such parental (environmental) effects play important roles in modifying offspring morphology, growth and reproduction (González et al., 2018; Dong et al., 2019b; Adomako et al., 2021; Wang et al., 2022; Yu et al., 2022). In particular, parental effects may induce offspring to make phenotypic adjustments to adapt to stressful environments that are similar to their parents, thereby conferring competitive advantages on plants (Baker et al., 2019; Puy et al., 2021a, b; Sobral and Sampedro, 2022).

Increasing studies have suggested that parental effects can transmit not only via sexual reproduction (sexual parental effects) but also via clonal propagation (clonal parental effects) (Latzel and Klimešová, 2010; Dong et al., 2019a; Luo et al., 2022; Yu et al., 2022; Zhang et al., 2023). For instance, light availability experienced by parental ramets significantly modified the morphology and physiology of their offspring ramets in *Wedelia trilobata* (Xiao et al., 2022). In *Alternanthera philoxeroides*, soil nutrient availability influenced parental ramets in ways that altered offspring growth performance (Wang et al., 2022). Similarly, insect herbivory altered both the growth and defense traits in offspring of *A. philoxeroides* (Dong et al., 2017). Moreover, parental exposure to copper stress shaped the tolerance of *Spirodela polyrhiza’*s offspring ramets to the same stress (Huber et al., 2021). These findings suggest that clonal parental effects can significantly shape offspring performance and, consequently, may influence competitive outcomes between neighboring plant species (Yu et al., 2022; Jin et al., 2023).

If clonal parental effects promote the growth of offspring ramets in a clonal plant species, they may substantially enhance the competitive ability of the offspring when competing with a heterospecific neighbor (Yu et al., 2022). In contrast, if such effects weaken the growth of offspring ramets of the clonal plant, they may reduce the offspring’s competitiveness (Yu et al., 2022). When two competing plant species are influenced in opposite ways, such as one benefiting while the other is suppressed, the competitive outcome may shift. Similarly, if both species are influenced in the same direction, whether positively or negatively, a significantly difference in the magnitude of clonal parental effects may still alter the competitive outcome between them.

Clonal parental effects on the offspring growth can vary with the environmental conditions that the offspring ramets currently face (González et al., 2017; Dong et al., 2018; Zhang et al., 2023). For example, when offspring ramets of *Trifolium repens* grew under drought conditions, there was no significant difference in their growth regardless of whether their parents had experienced drought or not (González et al., 2017). However, under control (non-drought) condition, offspring from drought-experienced parents showed reduced growth compared to those from control parents (González et al., 2017). Similarly, when clonal offspring of *A. philoxeroides* grew under high nutrient conditions, they performed better if their parents had grown under high rather than low nutrient condition (Dong et al., 2018). However, such a clonal parental effect diminished when clonal offspring were grown under low nutrient conditions (Dong et al., 2018). Thus, clonal parental effects on interspecific competition may also vary with the environmental conditions of offspring ramets.

To test the role of clonal parental effects in interspecific competition, we conducted a two-phase experiment using two clonal floating duckweed species, *Spirodela polyrhiza* and *Lemna minor*. We chose these two species because they often coexist and compete with each other, and also because they can propagate rapidly through clonal growth (Jin et al., 2021; Peng et al., 2023; Liu et al., 2024). In the parental phase, *S. polyrhiza* and *L. minor* were grown alone under two distinct nutrient conditions and produced offspring ramets. In the offspring phase, offspring ramets produced from parental phase were grown with or without a heterospecific neighbor (i.e., with or without interspecific competition) under the same two nutrient conditions. Specifically, we tested the hypothesis that clonal parental effects can alter the competitive interaction between the two duckweed species and such an effect may vary with the environment in which the offspring grow.

## Materials and methods

### Plant species

*Spirodela polyrhiza* (L.) Schleid and *Lemna minor* L., known as duckweeds, are both perennial aquatic clonal floating plants of the Araceae family. Duckweed species represent some of the simplest and smallest flowering plants, and commonly found in freshwater ecosystems of tropical, subtropical and temperate regions, such as rice paddies, ditches, and eutrophic still waters like lakes and ponds (Jacobs, 1947; Hillman, 1961). Duckweed species can rapidly produce new individuals (ramets) through clonal propagation (Zhang et al., 2023).

*S. polyrhiza* has flat, broad-ovate fronds measuring 5–10 mm in length and 3–8 mm in width, with adventitious roots emerging from the center of the abaxial surface of the frond. New buds form near the root base, develop into new fronds connected by a slender petiole, and eventually detach to form independent individuals (Lemon and Posluszny, 2000). Each ramet of *S. polyrhiza* consists of 2–3 fronds and adventitious roots. *L. minor* has nearly circular or broadly ovate fronds, green on the adaxial surface and light yellow or purple on the abaxial surface. The fronds measure 1.5–6 mm in length and 2–3 mm in width. A single filamentous root of 3–4 cm arises from the abaxial surface of the frond. New fronds form in a pouch on one side of the parent frond and detach once fully developed. *S. polyrhiza* and *L. minor* often co-occur in natural aquatic ecosystems, forming floating plant communities that covering water surfaces (Hillman, 1961; Liu et al., 2024).

### Sampling and cultivation

The original plant materials for this experiment were collected from a small area (~ 6 m^2^) of a slow-flowing stream in Jiaojiang District, Taizhou City, Zhejiang Province, China (28°3’N, 121°21′E), and subsequently cultivated in a greenhouse at the Jiaojiang Campus of Taizhou University (Jin et al., 2023). By collecting plants from a single, localized site, we aimed to minimize the potential influence of genetic variation. The collected plants were sterilized with 0.01 M NaClO for 30 s and rinsed twice with double-distilled water to minimize microbial contamination (Xing et al., 2010). Prior to the experiment, we cultured new ramets in plastic containers (64 cm long × 42 cm wide × 14 cm high) filled with 10% Hoagland solution (Srivastava et al., 2006).

### Experiment design

**Figure 1:**
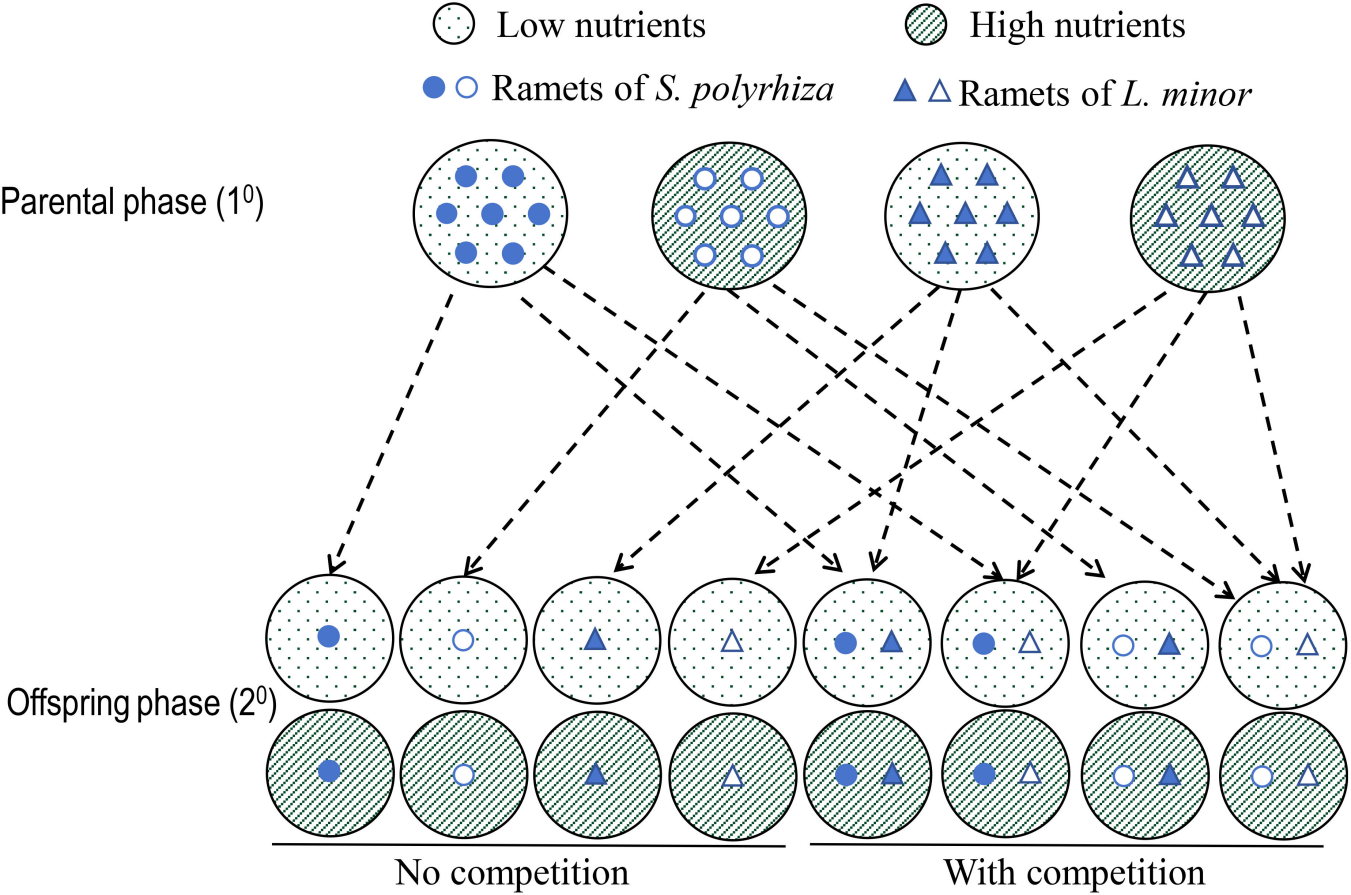
Schematic diagram of experimental design. In the parental phase (1^0^), each of the two duckweed species (*Spirodela polyrhiza* and *Lemna minor*) was grown alone at two nutrient levels (low and high) and each container (big circle) initially contained eight parental ramets of the same species. In the offspring phase (2^0^), each of the four types of offspring ramets produced in the parental phase was grown alone (no competition, one offspring ramet per container) or mixed with one type of the offspring of the other species (with competition, two offspring ramets per container) at both the high and low nutrient level.

This experiment consisted of two phases: the parental phase and the offspring phase. The parental phase (1^0^) involved two nutrient levels (low *vs*. high, i.e., 1/16× Hoagland solution *vs*. 1× Hoagland solution) and two plant species (*S. polyrhiza* and *L. minor*; **Figure 1**). The two species were cultured separately in the two nutrient levels, with ten replicates for each treatment, 40 containers in total. Each container (1.5 L; 17.5 cm in diameter and 10.5 cm in height) was filled with 1 L of the nutrient solution and initially contained eight ramets of the same species. The containers were randomly placed on a bench in a greenhouse, and the nutrient solutions were replaced every six days. This phase started on May 25, 2021, lasted for 16 days, and ended on June 5 when ramets in the containers covered the entire water surface. The average temperature in the greenhouse during the experiment was 28.1°C, with a relative humidity of 82% (iButton DS1923; Maxim Integrated Products, USA). One portion of the offspring ramets formed in this phase were used for the offspring phase, and the other portion was harvested to measure biomass (oven-drying at 70°C to constant weight) and number of ramets. Due to the incidence of insect herbivory, a total of 34 containers were harvested.

The competition treatments in the offspring phase (2^0^) used an additive design (**Figure 1**). During this phase, ramets were randomly selected from each parental treatment group. For the treatments with no competition, one offspring ramet of either species was grown in a container (12 cm in diameter and 6.5 cm in height) filled with 400 mL of a nutrient solution. Specifically, for each species, each of the two types of offspring ramets produced in the parental phase (i.e., offspring produced by the parent grown in the high and the low nutrient level) was grown alone at both the high and the low nutrient level as used in the parental phase (**Figure 1**). This resulted in eight treatments and 128 containers (each was replicated 16 times). For the competition treatments, one offspring ramet of both species was grown in a pot (**Figure 1**). All four mixtures of the two types of offspring ramets of the two species were grown at both the high and the low nutrient level, resulting in also eight treatments and 128 containers (16 replicates each). All 256 containers were randomly placed on a bench in the same greenhouse. This phase started on June 5, 2020, lasted for 21 days, and ended on June 26. The nutrient solutions were replaced every six days, and this phase ended when the ramets in the low nutrient level with competition covered the entire water surface in the containers. The average temperature in the greenhouse during this period was 27.9°C, with a relative humidity of 82%. Due to the incidence of insect herbivory, a total of 178 containers were harvested. We measured biomass (oven-drying at 70°C to constant weight) and number of ramets of each species.

### Harvest and measurements

At the harvest of each phase, ramets of *S. polyrhiza* and *L. minor* in each container were counted separately. Then, biomass was measured after oven-drying at 70°C to constant weight. Due to damage by insects, plants in 34 containers were finally harvested at the end of the parental phase and plants in 178 containers were harvested at the end of the offspring phase.

### Data analysis

For the data from the parental phase of the experiment, independent *t-tests* were used to assess the impact of nutrient availability on biomass and ramet number of each species. For the data from the offspring phase of the experiment, we first quantified interspecific competition intensity by calculating log response ratio (LogRR) for each species in each of the eight treatments (2 offspring nutrient levels × 2 parental nutrient levels of the target species × 2 parental nutrient levels of the competitor species): LogRR = Log (B*_i_*/B*_0_*), where B*_i_* represents biomass (or number of ramets) of the target species in the presence of the competitor species (with competition) of replicate *i*, and B*_0_* represents biomass (or number of ramets) of the target species in the absence of the competitor species (no competition) averaged across the replicates. LogRR has been widely used in previous studies to quantify the intensity of interspecific interactions (Bartelheimer et al., 2010; Si et al., 2019; Yu et al., 2022; Zhang et al., 2022a; Douce et al., 2023). It allows for the standardized comparison of performance with and without competitors (Goldberg et al., 1999). The negative values of LogRR indicate competitive effects, with smaller (more negative) values corresponding to stronger interspecific competition, i.e., greater suppression of this species by the other species (Zhang et al., 2022a; Peng et al., 2023).

We used three-way ANOVAs to test the effects of nutrient availability of the offspring ramets (2^0^ nutrients), nutrient availability of the parent of the target species (Target 1^0^), nutrient availability of the parent of the competitor species (Competitor 1^0^) and their interactions on LogRR of each of the two species. Data were square-root transformed when necessary to meet the assumptions of normality and homoscedasticity. To test whether the replicates that experienced obvious insect herbivory (and were excluded from the ANOVA analyses) were associated with treatment effects, we fitted binomial logistic regression models with herbivory status as the response variable and treatments as predictors. All data were analyzed using SPSS 22.0 software (IBM Corp., Armonk, NY, USA). The significance level was set at *P* < 0.05.

## Results

### Growth performance of parent plants

**Figure 2:**
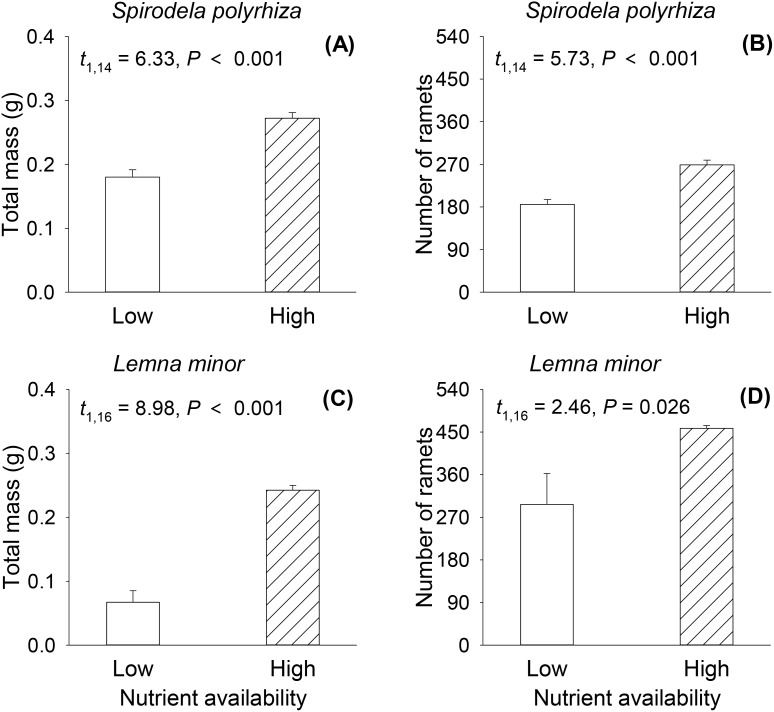
Effects of nutrient availability on total mass and number of ramets per container of the parental populations of *Spirodela polyrhiza* **(A, B)** and *Lemna minor* **(C, D)**. Bars and vertical lines represent mean and SE. *P-*, *t-*value and degree of freedom of *t*-tests are also given.

Nutrient availability significantly affected biomass and number of ramets of both duckweed species (**Figure 2**). Both total biomass and number of ramets of *S. polyrhiza* and *L. minor* were higher under high than under low nutrient availability (**Figures 2A-D**).

### Clonal parental effects on offspring competitive ability of *S. polyrhiza*

**Figure 3:**
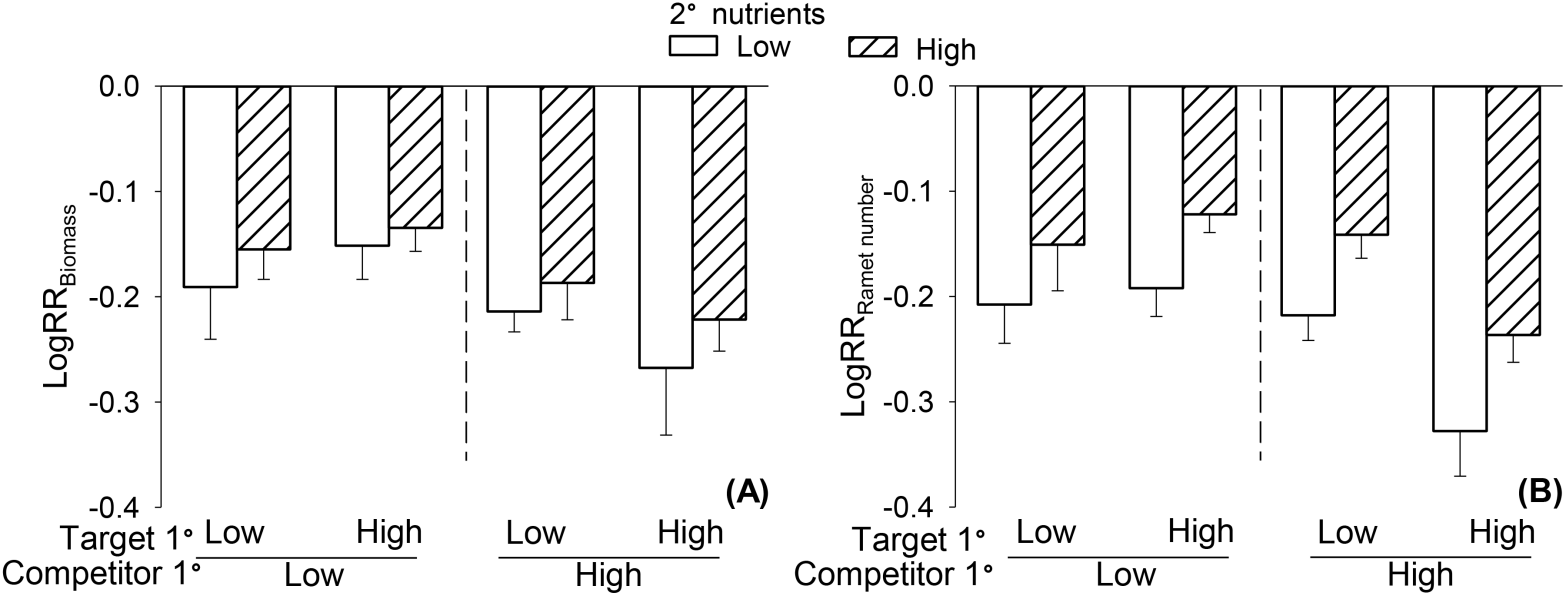
Effects of nutrient availability of offspring ramets (2^0^ nutrients), nutrient availability of the parent plants of *Spirodela polyrhiza* (Target 1^0^) and nutrient availability of the parent plants of *Lemna minor* (Competitor 1^0^) on offspring competitive ability of *S. polyrhiza*, as measured by log response ratio of biomass (LogRR_Biomass_, **A**) and ramet number (LogRR_Ramet number_, **B**). Bars and vertical lines represent mean and SE.

Nutrient availability experienced by the parent plants of *L. minor* significantly influenced the offspring competitive ability of *S. polyrhiza*, as indicated by both LogRR of biomass and ramet number (**Table 1**). Specifically, offspring competitive ability of *S. polyrhiza* was significantly less negative (i.e., higher) when the parent of *L. minor* (Competitor 1^0^) was grown under low rather than high nutrient conditions (**Figure 3**), indicating enhanced offspring performance of *S. polyrhiza* when its competitor’s parent experienced low nutrient conditions. For biomass, when the competitor’s parent was grown under high nutrient conditions, the competitive ability of the offspring population of *S. polyrhiza* was lower (**Table 1A**, **Figure 3A**). However, this effect on the LogRR of ramet number varied significantly depending on the nutrient availability experienced by the parent of *S. polyrhiza*, such that the clonal parental effect of *L. minor* was evident only when the parent of *S. polyrhiza* (Target 1^0^) was grown under high nutrient conditions (**Table 1B**, **Figure 3B**). Additionally, nutrient availability experienced by the offspring ramets significantly affected the LogRR of ramet number (**Table 1B**, **Figure 3B**).

**Table 1:** ANOVA results for effects of nutrient availability of offspring ramets (2^0^ nutrients), nutrient availability of the parent plants of *Spirodela polyrhiza* (Target 1^0^) and nutrient availability of the parent plants of *Lemna minor* (Competitor 1^0^) on offspring competitiveness of *S. polyrhiza*, as measured by log response ratio of biomass (LogRR_Biomass_, A) and ramet number (LogRR_Ramet number_, B).

Effect	df	(A) LogRR_Biomass_			(B) LogRR_Ramet number_		
		*F*	*P*	Partial *η*²	*F*	*P*	Partial *η*²
2^0^ nutrients (N)	1, 78	0.86	0.356	0.01	**10.37**	**0.002**	**0.12**
Target 1^0^ (T)	1, 78	0.04	0.848	<0.01	2.72	0.103	0.03
Competitor 1^0^ (C)	1, 78	**8.23**	**0.005**	**0.1**	**7.87**	**0.006**	**0.09**
N × T	1, 78	0.05	0.829	<0.01	0.14	0.710	<0.01
T × C	1, 78	2.07	0.154	0.03	**9.46**	**0.003**	**0.11**
N × C	1, 78	0.16	0.688	<0.01	0.39	0.536	<0.01
N × T × C	1, 78	0.01	0.938	<0.01	0.02	0.900	<0.01

Numbers are in bold when *P* < 0.05.

### Clonal parental effects on offspring competitive ability of *L. minor*

**Figure 4:**
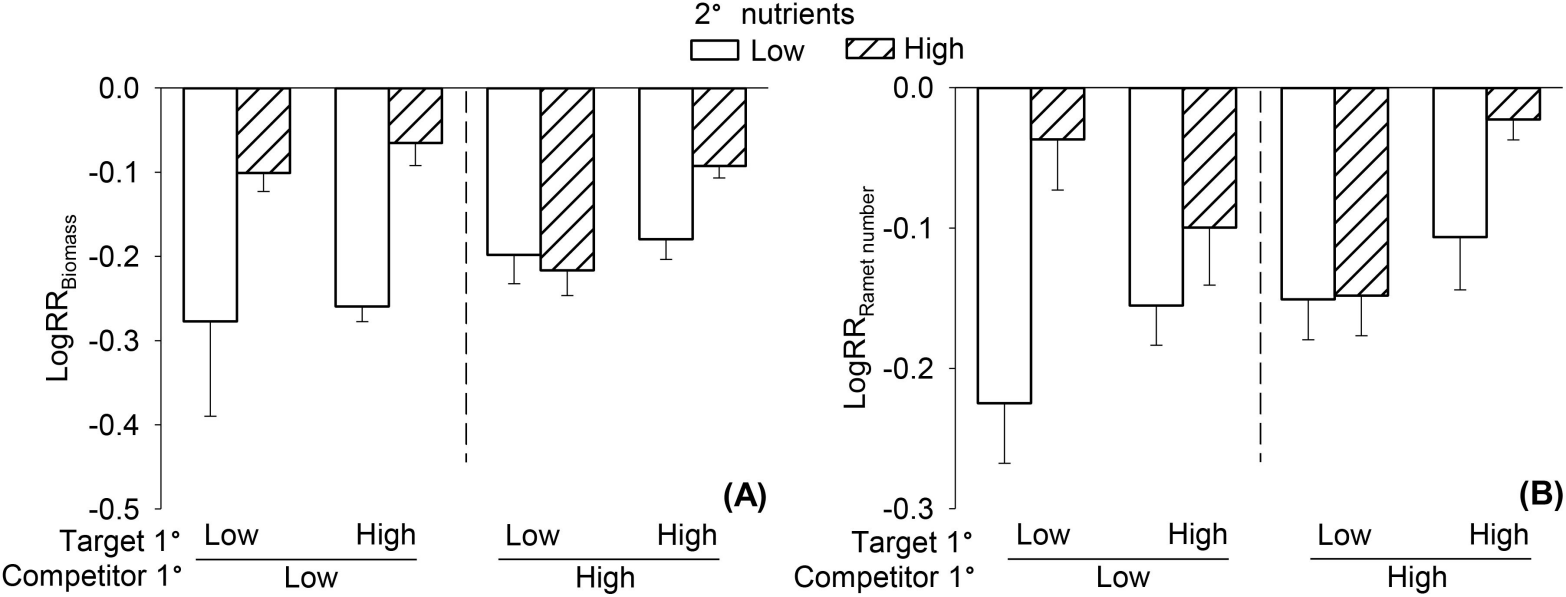
Effects of nutrient availability of offspring ramets (2^0^ nutrients), nutrient availability of the parent plants of *Lemna minor* (Target 1^0^) and nutrient availability of the parent plants of *Spirodela polyrhiza* (Competitor 1^0^) on offspring competitive ability of *L. minor*, as measured by log response ratio of biomass (LogRR_Biomass_, **A**) and ramet number (LogRR_Ramet number_, **B**). Bars and vertical lines represent mean and SE.

Nutrient availability of the offspring significantly influenced the competitive ability of *L. minor* (**Table 2**). The competitive ability of *L. minor* was significantly less negative (i.e., higher) when the offspring was grown under high rather than low nutrient conditions (**Figure 4**). However, this effect on the LogRR of biomass varied significantly depending on the nutrient availability experienced by the competitor’s parent. When the offspring was grown under low nutrient conditions, the competitive ability of *L. minor* was lower if the parent of *S. polyrhiza* had also experienced low nutrient conditions, whereas this trend was reversed under high nutrient conditions for the offspring (**Table 2A**, **Figure 4A**). Furthermore, a significant three-way interaction on the LogRR of ramet number indicated that nutrient availability experienced by both parental generations and the offspring jointly influenced the competitive ability of *L. minor*. When both the parent plants of *L. minor* and the parent plants of *S. polyrhiza* experienced high nutrient availability, the competitive ability of *L. minor* was highest under high nutrient conditions for the offspring (**Table 2B**, **Figure 4B**).

**Table 2:** ANOVA results for effects of nutrient availability of offspring ramets (2^0^ nutrients), nutrient availability of the parent plants of *Lemna minor* (Target 1^0^) and nutrient availability of the parent plants of *Spirodela polyrhiza* (Competitor 1^0^) on offspring competitiveness of *L. minor*, as measured by log response ratio of biomass (LogRR_Biomass_, A) and ramet number (LogRR_Ramet number_, B).

Effect	df	(A) LogRR_Biomass_			(B) LogRR_Ramet number_		
		*F*	*P*	Partial *η*²	*F*	*P*	Partial *η*²
2^0^ nutrients (N)	1, 78	**9.99**	**0.002**	**0.11**	**11.50**	**0.001**	**0.13**
Target 1^0^ (T)	1, 78	1.99	0.162	0.03	3.29	0.074	0.04
Competitor 1^0^ (C)	1, 78	0.01	0.909	<0.01	0.83	0.366	0.01
N × T	1, 78	0.78	0.379	0.01	0.28	0.601	<0.01
T × C	1, 78	0.41	0.522	<0.01	2.80	0.098	0.04
N × C	1, 78	**4.72**	**0.033**	**0.06**	2.61	0.110	0.03
N × T × C	1, 78	0.40	0.531	<0.01	**4.82**	**0.031**	**0.06**

Numbers are in bold when *P* < 0.05.

## Discussion

Our study demonstrates that nutrient conditions experienced by parent plants significantly affect the competitive performance of their clonal offspring in two duckweed species. While consistent with the general notion that parental environments affect offspring traits and growth (Dong et al., 2018; Huber et al., 2021; Wang et al., 2022), our findings further reveal that the magnitude and direction of clonal parental effects differ between species and are also influenced by the nutrient conditions experienced by the offspring. These results underscore the species-specific and context-dependent nature of clonal parental effects on offspring interspecific competition.

In this study, we found that the competitive ability of *S. polyrhiza* was influenced by the nutrient conditions experienced by the parent of its competitor. Specifically, *S. polyrhiza* showed higher competitive ability when grown with *L. minor* whose parent had experienced low nutrient availability, and this effect was independent of the nutrient conditions of the offspring. Such clonal parental effect on competition may be explained by a condition transfer mechanism, commonly referred to as the “silver spoon effect” (Grafen, 1988; Bonduriansky and Crean, 2018; Walsh et al., 2024). It occurs when parents experiencing favorable conditions produce offspring with enhanced fitness, regardless of the offspring’s environmental conditions. As the competitor (*L. minor*) parent was likely to produce smaller offspring ramets under low than under high nutrient availability, the suppressive effect of these offspring on the offspring of *S. polyrhiza* became weaker. Consequently, the competitive ability of *S. polyrhiza* was altered by the nutrient conditions of the competitor’s parent.

For *L. minor*, the competitive ability of its offspring was also significantly influenced by the nutrient condition of parent plants. However, the magnitude of this clonal parental effect varied with the nutrient condition of the offspring. While parental environments can shape offspring performance, the offspring’s own environment also plays a crucial role in determining their phenotypic traits (Latzel et al., 2013; González et al., 2017; Baker et al., 2018). Our study thus provides evidence that clonal parental effects on interspecific competition can vary with the environmental conditions of the offspring. In particular, when offspring grew under high nutrient availability, the competitive ability of *L. minor* was higher when its competitor’s parent grew under low than when it grew under high nutrient availability. Similarly, this clonal parental effect can also be explained by the silver spoon effect, whereby parent plants grown under low nutrient conditions produce smaller offspring with reduced competitive ability (Dong et al., 2019a; Wang et al., 2022; Zhang et al., 2022b). This reflects a form of parental provisioning, in which the quality of the parental environment determines the resources passed to offspring.

However, when offspring grew under low nutrient availability, the competitive ability of *L. minor* was lower when its competitor’s parent had grown under low nutrients than when it had grown under high nutrients. Previous studies suggest that higher parental provisioning does not always enhance the performance of clonal offspring, as it may be influenced by both the allocation of biomass to roots and the size (e.g., biomass) of the offspring ramets (Zhang et al., 2022b). According to the optimal partitioning theory (Bloom et al., 1985; Hilbert, 1990), plants allocate more biomass to roots when grown under low nutrient conditions. Based on this concept, we hypothesized that environmental plasticity in biomass allocation, induced by low nutrient availability, would enhance root-to-shoot ratio of offspring through clonal parental effects, particularly in the competitive species *S. polyrhiza*. Due to limitations of the duckweeds in this experiment, separating the roots of numerous small individuals was challenging. Since root plasticity plays an important role in nutrient acquisition and competitive interactions (Schiffers et al., 2011), the absence of root-related data weakens the interpretation of competition dynamics in this study. Future studies should incorporate root trait measurements to elucidate the role of root plasticity in clonal parental effects under different nutrient environments. Another possible explanation is that anticipatory parental effects of *S. polyrhiza*, wherein parental exposure to specific environments primes offspring to cope with similar conditions, enhancing their ability to face environmental challenges (Marshall and Uller, 2007; Walsh et al., 2024). While this anticipatory effect did not manifest as strongly in *L. minor*, the condition transfer effect appeared to modulate its competitiveness more than the anticipatory parental effect. Our results indicate that *L. minor* and *S. polyrhiza* exhibit different mechanisms of clonal parental effects on the competitive abilities of their offspring. It is worth noting that we hypothesize the observed parental effects may be driven by different mechanisms, such as parental provisioning and anticipatory parental effects (Grafen, 1988; Dong et al., 2019a; Walsh et al., 2024). However, we acknowledge that these interpretations are based on theories and previous studies, and further research integrating physiological and gene expression data is needed to validate them.

Our results suggest that clonal parental effects can influence the competitive ability of offspring and that this influence may vary depending on the offspring environment. Despite the potential for residual genetic variation among parental individuals, the observed effects in this study likely exceed what genotypic variation could be attributed to within the parental generation. In addition, one limitation of our study concerns the impact of insect herbivory. Insect feeding unexpectedly affected the survival and growth of both duckweed species in this study. Nonetheless, the regression analysis showed that the exclusion of replicates due to insect herbivory was not treatment-dependent, suggesting that our findings remain robust despite this exclusion. However, given that herbivory is common in natural settings and may be species-specific, it has the potential to influence the outcomes of interspecific competition (Center et al., 2005; Huang et al., 2014; Aschehoug et al., 2016; Subramanian and Turcotte, 2020). Therefore, while our findings suggest that parental effects can influence offspring competitiveness, this conclusion should be interpreted in the context of insect herbivory having been excluded from the analysis. Previous studies have shown that herbivory can significantly reduce the growth and reproduction of duckweed (Mariani et al., 2020) and other floating plants, such as *Eichhornia crassipes* (Center et al., 2005). Considering that natural ecosystems involve multiple types of interactions, including intra- and interspecific competition as well as herbivory and other biotic factors, future studies should explicitly include insect herbivory as an experimental factor. This would provide a more comprehensive understanding of how parental effects interact with biotic pressures to shape plant performance and competitive outcomes in aquatic ecosystems (Böttner et al., 2025).

## Conclusions

We conclude that clonal parental effects can influence the competitive ability of clonal plants, but the strength of this influence depends on the environmental conditions experienced by the offspring. Our findings underscore the role of clonal parental effects in mediating interspecific competition, with potential consequences for species composition and ecosystem productivity. Future research could explore how genetic variation, life history, and environmental factors combine to mediate these effects and contribute to the ecological and evolutionary success of clonal species.

## Data Availability

The raw data supporting the conclusions of this article will be made available by the authors, without undue reservation.

## Appendix

**Table A1:** Effects of treatments on the exclusion of replicates due to obvious herbivory based on logistic regression analysis.

Effect	Estimate	SE	z	*P*
*Spirodela polyrhiza*				
(Intercept)	0.8057	0.3825	2.106	0.0352
2^0^ nutrients	0.2896	0.3815	0.759	0.4477
Target 1^0^	-0.5755	0.3829	-1.503	0.1328
Competitor 1^0^	0.1451	0.3811	0.381	0.7035
*Lemna minor*				
(Intercept)	0.8057	0.3825	2.106	0.0352
2^0^ nutrients	0.2896	0.3815	0.759	0.4477
Target 1^0^	0.1451	0.3811	0.381	0.7035
Competitor 1^0^	-0.5755	0.3829	-1.503	0.1328
